# Development of the QoLISSY 0–4 questionnaire: a health-related quality of life tool for young children with short stature

**DOI:** 10.1186/s41687-025-00925-x

**Published:** 2025-07-25

**Authors:** Adekunle Adedeji, Stefanie Witt, Julia Quitmann

**Affiliations:** 1 Department of Social Work, Hamburg University of Applied Sciences, Hamburg, Germany; 2 Department of Medical Psychology, University Medical Center, Hamburg-Eppendorf, Hamburg, Germany

**Keywords:** Health-related quality of life (HRQoL), Short stature, Pediatric assessment, Questionnaire development, Cognitive debriefing, Early childhood health, QOLISSY, PROMs

## Abstract

Short stature in children aged 0–4 years presents unique physical, social, and emotional challenges that significantly impact health-related quality of life (HRQoL). The QoLISSY 0–4 questionnaire was developed as a Patient Reported Outcome measure (PROMS) to address the absence of an age-specific, condition-focused HRQoL assessment tool for this population. A mixed-method approach was employed to adapt the original QoLISSY questionnaire for children aged 5–18. Qualitative interviews were conducted with 24 parents of children diagnosed with short-stature conditions, including achondroplasia, small for gestational age, growth hormone deficiency, and Silver-Russell Syndrome. Cognitive debriefing sessions and iterative feedback guided the development of new items tailored to the needs of children aged 0–4. Pilot testing involved 20 parents, who evaluated the questionnaire’s clarity, relevance, and comprehensiveness. The development process yielded an 8-domain, 55-item questionnaire addressing physical health, social interactions, emotions, coping mechanisms, medical care, future concerns, and parental impact. Cognitive debriefing results indicated high item clarity (100%), relevance (93%), and importance (94%), with parents confirming that the questionnaire effectively captured their child’s HRQoL experiences. The QoLISSY 0–4 questionnaire provides a tailored, parent-reported tool for assessing HRQoL in children aged 0–4 with short stature. Its development reflects a rigorous, participant-informed process ensuring its relevance and usability. Future validation studies will explore its cross-cultural applicability and psychometric properties to establish its utility in research and clinical settings.

## Background

Health-related Quality of Life (HRQoL) assessment has proven crucial in understanding the broader impact of medical conditions on patients and their families, particularly during childhood. HrQoL encompasses not only the physical aspects of health but also emotional, social, and psychological well-being, which can be profoundly affected by chronic or congenital conditions [1]. In pediatric care, measuring HRQoL provides valuable insights into how a child’s health condition influences their daily life, development, and overall sense of well-being, as well as how it affects family dynamics, caregiving demands, and parental stress [2].

For children with conditions such as achondroplasia, small for gestational age, growth hormone deficiency, and Silver-Russell Syndrome, short stature is often accompanied by a range of health challenges that go beyond physical growth. These conditions can impact mobility, cognitive development, and social interactions, reducing HRQoL [3]. The complexity of HRQoL in relation to short stature conditions further underscores its usefulness for understanding the full scope of how a health condition affects a child’s physical, emotional, and social well-being, especially in early childhood. An accurate understanding of this interplay enables healthcare providers to tailor interventions that address medical needs and broader developmental and psychological challenges [4]. Furthermore, capturing HRQoL using Patient-Reported Outcome Measures (PROMs) with condition-specific measures allows focus on the unique experiences and challenges associated with a particular condition, providing a more precise and relevant assessment of how it affects an individual’s life. For example, short-stature children may experience difficulties with physical tasks due to growth or face psychological distress due to delayed development relative to their peers [5].

This need for accurate, condition-specific HrQoL measurement led to the development of the QoLISSY (Quality of Life in Short Stature Youth) questionnaire, designed for children aged 5 to 18 years with short stature. The QoLISSY instrument has been validated as a reliable tool for assessing the HRQoL in older children, capturing the diverse challenges these conditions present in adolescence and early childhood [6, 7]. It provides insights into physical limitations, emotional distress, and social difficulties faced by children in this age group, empowering healthcare providers to better understand and address these issues. However, while the QoLISSY questionnaire fills an essential gap for school-aged children and adolescents, there remains a critical need for an age-specific HRQoL instrument tailored to younger children, particularly those aged 0 to 4. Early childhood is a formative stage where developmental milestones are reached, and the effects of medical conditions can shape long-term physical and emotional well-being [8].

### Short stature in children aged 0–4 years: clinical and developmental considerations

Short stature in early childhood (0–4 years) presents distinct clinical and developmental challenges. This period is critical for physical growth, cognitive development, and social integration. Conditions such as achondroplasia, small for gestational age (SGA), Silver-Russell syndrome (SRS), and growth hormone deficiency (GHD) can significantly impair growth trajectories and developmental outcomes [9].

Achondroplasia is a genetic disorder that affects endochondral ossification, resulting in disproportionate short stature, macrocephaly, and limb shortening. Associated complications—such as spinal stenosis and respiratory issues—can emerge in infancy, potentially delaying motor and social development [10]. SGA infants, often due to intrauterine growth restriction, remain at risk for persistent growth failure and psychosocial difficulties. These children may struggle with peer integration and self-esteem during early socialisation phases [11]. SRS is characterised by prenatal and postnatal growth retardation, facial dysmorphism, and feeding difficulties. These features necessitate early nutritional and therapeutic interventions to support growth and mitigate developmental delays [12]. GHD results in insufficient growth hormone production, which impairs linear growth and can potentially affect metabolism, emotional regulation, and social functioning. Early diagnosis and treatment are essential to support normal development [13].

Given their limited ability to express symptoms, children in this age group rely heavily on caregiver observations. Parental insights into physical, emotional, and social functioning are vital for assessing health-related quality of life (HRQoL) and guiding early interventions [14].

### The current study

The QoLISSY questionnaire was developed to provide a short stature-specific quality of life assessment for children aged 5 to 18. However, despite the importance of early childhood in shaping later development and well-being, no validated instrument has yet been adapted from the parental perspective for younger children (0–4 years). The current study aims to fill this gap by developing and pilot-testing a version of the QoLISSY questionnaire tailored to the needs of children aged 0 to 4 with ACH, SGA, SRS and GHD. By focusing on the parental perspective, the study aims to capture the early childhood experiences associated with these conditions and provide a reliable and valid tool to assess the quality of life in this vulnerable age group.

## Methods

### Study design

A mixed-method approach was adopted to assess the adaptability of the QoLISSY questionnaire and develop new items for children in this age group. The process involved conducting qualitative interviews with 24 parents of children with various diagnoses and administering a paper version of the original QoLISSY. The results from both qualitative and quantitative approaches facilitated the development of a version of the QoLISSY questionnaire tailored for parents of children aged 0–4. This newly developed version was then pilot-tested, followed by a cognitive debriefing to refine the items.

### Study population

From a total of N = 30 contacts, the study involved the parents of 24 children, all aged 0 to 4 years, who had been diagnosed with short-stature conditions. The distribution by diagnosis included 17 parents of children with Achondroplasia, two parents of children with SGA, three parents of children with SRS and two parents of children with GHD. These parents provided valuable insights into how these conditions affected their children’s physical development, social interactions, and overall quality of life during this critical early childhood stage.

### Recruitment and procedure

**Fig. 1:**
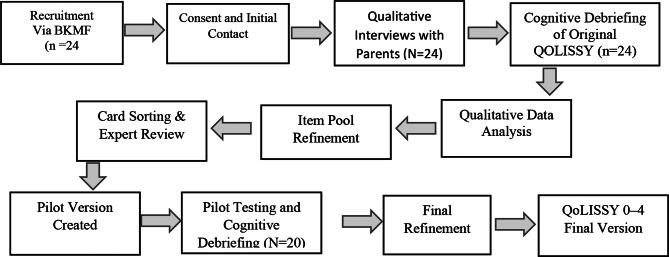
Study procedure flow chart

Participants were recruited through the patient organisation, *Bundesverband Kleinwüchsige Menschen und ihre Familien e.V. (BKMF)*. Study information was made available to parents, who were encouraged to contact the Quality of Life Research group at the Institute and Polyclinic for Medical Psychology, University Medical Centre Hamburg-Eppendorf, Hamburg, Germany. After initial contact via telephone or email, participants received a brief explanation of the study’s purpose and were asked to provide their mailing address. A detailed study information sheet and consent forms were then sent to them, along with a return envelope. Upon receiving consent, an appointment for a telephone interview was arranged, and a paper questionnaire, together with cognitive debriefing materials, was sent by post. Participants were required to complete the questionnaire before the interview and return it afterwards. Upon completing this study phase, a 50-euro gift card was awarded to each participant (see Fig. 1).

For pilot testing, the refined questionnaire, along with cognitive debriefing for each item, was posted to all participating parents. The questionnaires were returned upon completion. Out of the 24 parents, 20 returned the completed questionnaire. Two parents were unreachable due to a change of address. Each participant received a 10-euro gift card as an incentive for participating in the pilot study.

### Study Phase 1: Interview and first cognitive debriefing of paper questionnaires

#### Interview

Twenty-four interviews were conducted online and via telephone between March 2023 and April 2024. These interviews provided a flexible and adaptable platform for participants to engage in subjective evaluations of their children’s HRQoL concerning their short stature [15, 16]. An interview guide was developed to effectively guide the discussions based on the categories in the original QOLISSY questionnaire while also reflecting broader aspects relevant to HRQoL. This guide covers general life and diagnosis topics, everyday life, social environment, mental health, medical care, and future worries. All interviews were conducted in German.

Open-ended questions were crafted to allow participants to express their views naturally, with follow-up prompts designed to encourage deeper reflection and conversation. The interviews were conducted by three research scientists, all of whom were trained in psychology. Each facilitator began the interview by introducing the purpose of the study using a standardised text, emphasising the confidentiality of the discussions and the importance of respectful listening. Participants were reassured that their perspectives were valuable and that there were no right or wrong answers.

During the interviews, interviewers employed active listening techniques. The interviews lasted an average of 45 minutes and were audio-recorded with participants’ consent to ensure accuracy in transcription and analysis. After the interviews, participants were debriefed, and their questions or concerns were addressed.

### First cognitive debriefing of paper questionnaires

The first paper cognitive debriefing included all items from the original version of the QoLISSY. The QoLISSY questionnaire is designed to evaluate the health-related quality of life (HRQoL) in children and adolescents with short stature and their families. It includes domains addressing physical health, social stigma, emotional well-being, and the broader family impact of managing growth-related conditions [6]. The inclusion of this questionnaire is justified by its diverse coverage of disease-specific domains. Despite its focus on children and youths from older age groups, the original QOLISSY questionnaire shares a unifying characteristic: It is designed for the pediatric population and emphasises aspects of well-being. This complementary approach ensures a holistic evaluation of the health-related quality of life and well-being in children and their families, enabling a nuanced understanding of individual experiences for children aged 0–4 across various short-stature conditions.

**Fig. 2:**
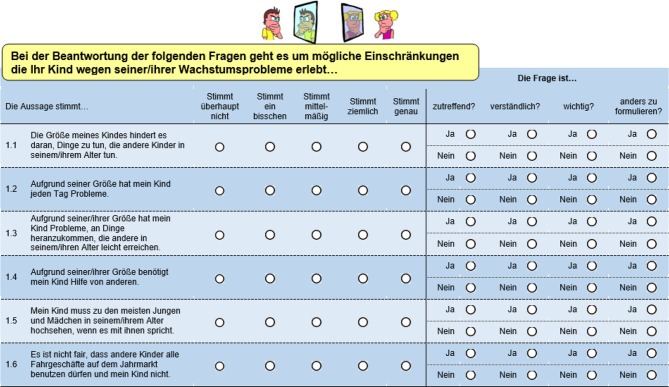
Presentation of items and first cognitive debriefing for original QOLISSY

The questionnaire was sent by post to each participant with a return envelope. Participants were required to answer every question and then answer additional questions on relevance, clarity, importance, and whether the item required rewording to fit the needs of children aged 0 − 4 from the parents’ perspectives. The questions were administered in the original study language (German) and presented in a matrix format, grouped by subdimension. An example is presented in Fig. 2 above.

At the end of the questionnaire, participants were asked to provide a general evaluation of the questionnaire in relation to the specific needs of short-stature children aged 0 to 4. They were prompted with specific questions, such as: How did you find filling out the questionnaire? Do you think the statements in the questionnaire reflect your child’s daily feelings or challenges? Do you believe the questionnaire topics relate to your child’s condition? Are there any important questions or issues about short stature that were not addressed?

### Study Phase 2: Pilot testing of the QOLISSY 0–4 questionnaire

The pilot testing of the QOLISSY 0–4 questionnaire aimed to assess the instrument’s clarity, relevance, and comprehensibility for parents of children with short stature. The questionnaire comprises eight categories, totalling 55 items. For the pilot phase, the questionnaire was sent by post to all 24 parents who participated in the initial phase of the study.

Cognitive debriefing questions were attached to the 55 items to ensure comprehensive feedback. These debriefing questions evaluated the clarity, relevance, importance, and whether the item required rewording. Each item was rated on a 5-point Likert scale, where 1 indicated “totally disagree” and 5 indicated “totally agree.” Each cognitive debriefing question was answered with a simple “yes” or “no.” For example, participants were asked, “Is this question clear?” to assess clarity.

An additional section containing quantitative and qualitative questions was included at the end of the questionnaire. This section consisted of four quantitative items assessing:

1. The ease of completing the questionnaire,
2. Whether the questionnaire addressed the everyday challenges of their child related to the diagnosis,
3. Whether the questionnaire appropriately captured the child’s feelings about living with short stature.
4. The completeness of the questionnaire

The qualitative component, which consisted of five open-ended questions, allowed participants to elaborate on their responses and explain why they chose specific answer options. The final question invited parents to provide general comments or suggestions for improvement regarding the overall content and format of the questionnaire. This structured approach to cognitive debriefing, combined with quantitative and qualitative feedback, ensured that the QOLISSY 0–4 questionnaire was thoroughly evaluated for usability, content relevance, and overall comprehensibility.

### Cross-linguistic item translation (German to English)

The English translation of the field questionnaire was undertaken to enhance its international applicability while ensuring cultural and linguistic accuracy. A rigorous, iterative translation process was conducted by two bilingual researchers, each of whom was fluent in both English and German. This process aims to preserve the original meaning and nuances of each item. Additionally, an AI-based tool was employed to refine the translation by generating suggestions, such as “suggest alternative simplifying words,” to improve clarity and accessibility. The researchers carefully reviewed and adjusted these AI-generated suggestions (using DeepL, an AI-based tool) to ensure that the translated items accurately captured the intended constructs and were suitable for diverse international contexts. However, it is essential to note that this version has not been tested for validity. The original German field scale is included in Appendix 1.

### Data analysis and item development

The development and evaluation of the QoLISSY 0–4 questionnaire were conducted in two key phases—item generation and pilot testing—using a mixed-methods approach that integrated qualitative and quantitative analyses.

### Phase 1: Qualitative analysis and item generation

To develop a condition- and age-specific instrument for assessing Health-related Quality of Life (HrQoL) in children aged 0–4 with short stature, qualitative interviews were conducted with 24 parents. All interviews were audio-recorded and transcribed using the intelligent verbatim technique to retain essential meaning while excluding extraneous speech fillers [17]. A deductive coding framework, based on the original QoLISSY domains, guided the analysis. Three trained coders independently identified recurring themes and child-specific concerns, which were then classified under preliminary HrQoL domains such as physical health, emotional well-being, social participation, and parental burden [18].

In parallel, a cognitive debriefing of the original QoLISSY items was conducted to assess their relevance, clarity, importance, and need for rewording when applied to the 0–4 age group. Open-ended comments provided by parents were analysed thematically to identify content gaps and opportunities for item refinement. Feedback emphasised the need for simpler language, toddler-specific examples, and new content areas such as sleep quality and accessibility in public spaces.

Insights from the interviews and cognitive debriefing were synthesised through a stepwise deductive process and second-level content analysis. Subcodes from the narratives were reviewed and transformed into candidate items, resulting in an initial pool of 98 items. Existing QoLISSY items deemed relevant were adapted for developmental appropriateness. The whole item pool underwent expert review and a card-sorting procedure [19] Involving three trained researchers in psychology and public health. The outcome was a refined 55-item pilot version organised into eight HRQoL domains: Physical Health, Social Interactions, Childcare, Emotions and Feelings, Coping Mechanisms, Medical Care, Future Concerns, and Impact on Parents.

### Phase 2: Quantitative and qualitative analysis – pilot testing

Pilot testing was conducted with 20 parents who had completed the earlier qualitative phase of the study. Each item in the 55-item instrument was rated on a 5-point Likert scale (1 = “totally disagree” to 5 = “totally agree”). In parallel, cognitive debriefing responses assessed each item’s clarity, importance, relevance, and need for rewording using yes/no questions.

Descriptive statistics (means, standard deviations, frequencies, and percentages) were calculated to assess item performance. Results demonstrated high levels of agreement across most items regarding clarity (100%), importance (94 % +), and relevance (93%). Only one item, “My child is irritable,” was flagged for rewording by one participant. Furthermore, four usability questions at the end of the questionnaire assessed ease of completion, coverage of daily challenges, emotional relevance, and perceived completeness. Over 93% of participants agreed that the questionnaire accurately captured the everyday realities and emotions of children with short stature, and 100% affirmed the instrument’s completeness.

Open-ended responses from the pilot phase were thematically analysed to identify suggestions for improvement. These included the addition of a new item addressing accessibility challenges in public spaces and the rewording of select items for improved comprehension. This qualitative feedback was crucial for refining the final instrument.

## Results

### Phase One: Cognitive debriefing analysis

Two cognitive debriefings were conducted —first, with the original QoLISSY items to assess their relevance and clarity for the 0–4 age group, and subsequently with the revised QOLISSY 0–4 version following item development. The first cognitive debriefing of the original QOLISSY questionnaire provided valuable insights into the clarity, importance, relevance, and wording of items assessing Health-Related Quality of Life (HRQoL) for children with short stature (see Table 1).

**Table 1:** Cognitive debriefing of original QOLISSY questionnaire for children aged 0–4 (percentage distribution) N = 24

	Strongly disagree	Disagree	Neither agree nor disagree	Agree	Strongly agree
Easy to complete the questionnaire.	-	18.2	36.4	45.5	-
Addressed the everyday challenges of their child related to the diagnosis.	-	-	9.1	45.5	45.5
Appropriately captured the child’s feelings about living with short stature.	-	-	9.1	45.5	45.5
The questionnaire is complete.	55.6	-	-	22.2	22.2

### Clarity of items

Participants offered significant feedback regarding the clarity of the questionnaire items. Only 12 out of the 65 items achieved a clarity score of 80% or above. Meanwhile, 46 items scored above 50%, and one item scored less than 30%. The overall average clarity score for the questionnaire was 60.5%, indicating that many items were perceived as ambiguous or unclear. For instance, participants highlighted that phrases such as “participation in physical activities” could be interpreted differently depending on the age of the children (0–4 years). These findings underscore the need for further refinement of item phrasing to enhance clarity.

### Importance of items

Participants evaluated the importance of each item in reflecting on their experiences. Five of the 65 items received an importance score above 80%, particularly those addressing emotional and social dimensions of HRQoL. Additionally, 40 items scored higher than 50%, with the questionnaire’s average importance score being 56.8%. Participants clarified that those items focusing on less frequently encountered scenarios (e.g., “situations with friends at school”) were perceived as less relevant to the daily lives of children aged 0–4 years. This feedback suggests that items should align more closely with the lived experiences of the target age group.

### Relevance of items

The relevance of each item to the HRQoL framework was critically assessed. Only 8 of the 65 items achieved an 80% or above relevance score. Furthermore, 20 scored above 50%, and 26 scored 30% or higher. The average relevance score for the original QOLISSY questionnaire for children aged 0–4 was 35.6%, indicating that most items did not fully reflect the concerns and experiences of parents of children with short stature. Participants also suggested including additional items to capture important aspects of daily life that were overlooked, such as sleep quality. These findings highlight the need to better align the questionnaire content with this population’s unique needs and challenges.

### Need for rewording

Analysis revealed that 58 of the 65 items require rewording to enhance comprehension and engagement. Participants recommended simplifying complex terms and phrases to improve accessibility for all respondents. Items containing medical jargon or technical terms were flagged as potential barriers to understanding. Simplification and the use of plain language are necessary to ensure the questionnaire is accessible and meaningful to its target audience.

Overall, the cognitive debriefing analysis identified significant areas for improvement in the QOLISSY questionnaire. Adjustments to clarity, relevance, and wording are essential to ensure the questionnaire accurately captures the experiences and concerns of parents of children aged 0–4 with short stature.

The findings from the cognitive debriefing indicate that the current items used in the original QOLISSY to assess HRQoL for children aged 0–4 are insufficiently capturing this age group’s unique needs and experiences. Only about 45% of the participants found it easy to complete the questionnaire. Similarly, about 65% of the participants expressed concerns that the original QOLISSY questionnaire did not adequately address the everyday challenges of their child concerning their age and short stature or specific developmental, emotional, and social contexts relevant to very young children. Furthermore, only 22% of the participants rated the original QOLISSY as complete in capturing the HrQOL of children aged 0–4 with short stature. The poor results emphasise the pressing need to develop new items that reflect the distinct challenges and concerns faced by children aged 0–4 with short stature. Additionally, feedback highlighted the importance of framing questions that align with this age group’s developmental milestones and language comprehension levels. Many parents indicated that the language used in the assessment tool was not always relatable or easily understood when discussing their children’s experiences.

### Item development for QOLISSY 0 – 4

The qualitative findings from parental interviews formed the foundation for generating content-rich and developmentally appropriate questionnaire items. Drawing on a stepwise deductive coding approach, key themes were identified across major domains of Health-related Quality of Life (HrQoL), which were then used to construct domains tailored to the experiences of children aged 0 to 4 with short stature. Each domain reflects a distinct aspect of the child’s lived reality, from physical limitations to emotional well-being and the broader impact on parental mental health. These themes were supported by illustrative quotations from parents, ensuring that the language and focus of each item aligned closely with the day-to-day experiences and challenges reported. The following section outlines the eight identified domains, each illustrated with an exemplary parental quote, which served as the empirical basis for item development.

### Physical health

This domain encompasses the physical limitations and health-related challenges children face due to their growth issues. Children with short stature may struggle with physical activities, resulting in difficulty participating in sports or other active play. These challenges can lead to a potential decline in overall fitness, contributing to feelings of frustration and exclusion among peers.

Example Quotation*: “We still have to carry < Child 11 >, of course. As I said, he is finally starting to walk while holding our hand. But mostly, he gets tired quickly, of course.”* (Person 11)

### Social interactions

This domain explores how children with short stature interact with their peers, family members, and strangers. Many children report feeling different from their peers, hindering their ability to form friendships and participate in group activities. Such social challenges can negatively affect their self-esteem and confidence.

Example Quotation: *“Yes, it is really about lugging him around. This intrusive behaviour: ‘I will just pick you up and take you somewhere, then put you down,’ even though he did not want to go there, right?”* (Person 1)

### Childcare

This domain examines how children cope with their size in childcare environments. It considers their interactions with caregivers and the degree to which these settings are accommodating. Some children may face difficulties due to inadequate facilities, such as furniture not appropriately sized for their needs, leading to frustration and discomfort.

Example Quotation*: “But what also happens, for example, and what I have observed quite often: especially the older girls at the kindergarten—since it goes up to six years until they start school, of course, like everyone else. Um, when he is standing somewhere and playing or daydreaming outside, the girls come along, pick him up like a doll, and carry him somewhere before setting him down.…”* (Person 1)

### Emotions and feelings

This domain focuses on children’s emotional responses to their growth condition. Children may experience various feelings, including frustration, sadness, and anxiety about their height and how it affects their daily lives. These emotions can significantly impact their mental well-being and overall quality of life.

Example Quotation: “*He does not like that. Or when other kids tell him, ‘You cannot do that, (child’s name), you are too small,’ he gets really stubborn. He sulks and then does it on purpose anyway. Be-/ because he understands everything—he is almost three, you know.”* (Person 1)

### Coping mechanisms

This domain assesses the strategies children use to manage their feelings and experiences related to their size. Coping mechanisms can be positive, such as seeking support from friends or family, and negative, like withdrawing from social situations. Understanding these coping strategies can provide insights into how children adapt to their challenges.

Example Quotation*: “Uh, so for (child’s name), his thing right now is always his pacifier. And then he always comes and says, ‘Mama, bed and pacifier.’ Then he needs about ten minutes to himself, takes his pacifier, lies down in bed, and after that, he is good to go again…”* (Person 3)

### Medical care

This domain examines the medical interventions and treatments that children receive for their growth disorders. It includes their experiences with healthcare providers, the effectiveness of treatments, and how these medical experiences influence their quality of life. Regular visits to healthcare professionals can be a source of anxiety for some children, who may have concerns about their treatment and future growth.

Example Quotation: *“I think it is simply the fear of what is coming. For example, measuring has been a big issue recently. They have to measure their arm span and legs, and there has just been so much fussing over him that he did not want, uh, and from strangers, too. I think it is just the fear.”* (Person 16)

### Future concerns

This domain captures the worries that parents and children have about the future. Concerns may revolve around long-term health, social acceptance, and the potential for growth-related challenges as they age. Both parents and children may wonder about the implications of short stature on future opportunities, including education and social relationships.

Example Quotation: *“Mhm (thoughtfully), well, I am also kind of ‘afraid,’ in quotation marks, of puberty. I think it will not be easy since it is never easy for any child. However, um, I think it will be especially difficult for her, in a completely different way than for other kids.”* (Person 15)

### Effect on parents

This domain explores how a child’s growth issues affect their parents’ emotional and psychological well-being. Parents may experience a range of feelings, including stress, anxiety, and helplessness concerning their child’s condition. The emotional burden of worrying about their child’s happiness and social acceptance can be significant.

Example Quotation: *“Yes, absolutely more support outside the home. And I think what would have helped us much earlier is psychological support. (…) Because that is not something that’s just handed to you or is easily available to you as parents. Instead, you pretty much only get it when, essentially, it is already too late for the child.”* (Person 17)

### Pilot testing and second cognitive debriefing QOLISSY 0 – 4

The pilot test results highlighted the need for minor revisions to some items, ensuring the questionnaire’s reliability and relevance. The feedback from both the quantitative ratings and qualitative responses was synthesised to guide final adjustments before moving on to larger-scale testing phases.

Table 2 shows that the pilot testing results of the QOLISSY 0–4 scale demonstrated significant improvements across all four parameters assessed during the cognitive debriefing process: importance, clarity, appropriateness, and wording. Notably, all items received an importance rating of at least 80%. Among the 56 items, 45 achieved an applicability score of 90% or higher, and 55 were rated with 100% clarity. However, one item, “< My child is irritable > *Mein Kind ist reizbar*,” was marked as unclear by a single participant. Additionally, 53 items attained an importance score of 94% or higher, while only one item fell below 94% for wording. When assessing the 0–4 questionnaire as a whole, 73% of participants reported that it was easy to complete, 93% felt the statements accurately described their child’s feelings or daily life challenges, 93% agreed that the topics in the questionnaire were relevant to their child’s illness, and 100% agreed to the completeness of the QOLISSY 0–4 regarding topics related to short stature they found important (see Table 3).

**Table 2:** Percentage distribution of second cognitive debriefing for pilot QOLISSY 0 − 4 with item translation in english (N = 20)

	Applicability (%)	Clarity (%)		Important (%)	Need Rewording (%)	
**Physical Health**						
Generally speaking, my child is healthy.	100	100		100	100	
My child suffers from physical pain related to his short stature.	100	100		100	100	
My child can get around in an age-appropriate way and explore the surroundings.	100	100		100	100	
My child has lots of energy.	100	100		100	100	
My child shows age-appropriate fine motor skills (e.g. grasping and holding small objects).	100	100		100	100	
My child shows age-appropriate gross motor skills (e.g. crawling, walking, jumping).	100	100		100	100	
My child has difficulty with food due to problems related to short stature.	94,1	100		94.1	100	
My child has difficulty sleeping due to problems related to short stature.	88.2	100		100	100	
My child’s ability to participate in daily activities is affected by problems related to short stature.	100	100		100	100	
**Social Domain**						
My child participates in social activities outside the daycare.	94.1	100		94.1	94.1	
My child has been treated unfairly by others due to their short stature.	94.1	100		94.1	94.1	
My child is limited in social activities due to short stature.	100	100		94.1	100	
My child is involved in family activity	100	100		94.1	100	
My child is supported by other members of the family	100	100		94.1	100	
Our living space is adapted to the physical abilities and needs of my child.	100	100		94.1	88,2	
My child is disadvantaged due to limited accessibility in public spaces.	Na	na		na	Na	
**Daycare**						
My child enjoys going to the daycare center.	100	100		94.1	100	
The staff at the daycare center are familiar with my child’s special needs.	94.1	100		100	100	
The daycare center provides special support for my child needs.	94.1	100		100	100	
The daycare center’s facilities are adapted to my child’s physical size.	100	100		94.1	100	
My child gets along well with other children at the daycare center.	100	100		94.1	100	
Other children at the daycare center take my child’s special needs into consideration.	82,4	100		88.2	100	
**Feelings and Emotions**						
My child is happy and content.	100	100		100	100	
My child is irritable.	82.4	94.1		88.2	94.1	
My child is frustrated.	94.1	100		94.1	100	
My child displays age-appropriate behaviour (e.g., playfulness, curiosity).	94.1	100		100	100	
My child is stressed.	82.4	100		94.1	100	
My child is anxious.	88.2	100		94.1	100	
**Coping**						
My child is frustrated due to physical limitations associated with short stature	82,4	100		94.1	100	
My child is able to effectively manage the limitations caused by their short stature.	100	100		100	100	
My child is confident when trying out new activities.	94.1	100		94.1	94.1	
My child is adaptable and participates in daily activities.	100	100		100	100	
My child reacts to changes positively.	100	100		100	100	
**Medical Domain**						
The frequency of doctor/therapist visits is exhausting for my child.	94.1	100		94.1	100	
My child is afraid of doctor visits.	94.1	100		94.1	100	
My child is afraid of medical examinations.	100	100		94.1	100	
My child is afraid of growth therapy.	82.4	100		94.1	100	
My child experiences emotional problems due to growth therapy.	82.4	100		94.1	94.1	
My child experiences physical problems due to growth therapy.	82.4	100		94.1	94.1	
Medical treatments negatively affect my child’s mood.	88.2	100		94.1	100	
Medical treatments negatively affect my child’s behaviour.	94.1	100		100	100	
My child has a positive attitude towards medical treatment.	100	100		100	100	
**Future Concerns**						
I am worried about my child’s future height.	100	100		100	100	
I am worried about my child’s physical development.	100	100		100	100	
I am worried about potential future health complications for my child.	100	100		100	100	
I am worried about whether my child will be accepted by society as they grow older.	100	100		100	100	
I am worried that my child might face academic challenges due to their height.	100	100		100	100	
I am worried about my child’s ability to become independent and take care of themselves.	100	100		100	100	
**Effects on parent**						
I feel emotionally overwhelmed by my child’s growth problems.	94.1	100		100	100	
I feel sad because of my child’s growth problems.	100	100		94.1	100	
I feel anxious because of my child’s growth problems.	94.1	100		100	94.1	
I suffer from fatigue and exhaustion due to my child’s special care needs.	94.1	100		94.1	100	
My child’s growth problems affect my leisure activities and social behaviour.	94.1	100		94.1	100	
I feel isolated because of my child’s growth problems.	88.2	100		88.2	100	
I feel misunderstood because of my child’s growth problems.	100	100		94.1	100	
The additional financial burden caused by my child’s unique needs due to their growth problems is difficult to manage.	94.1	100		100	100	
I feel prepared for future challenges arising from my child’s growth problems.	100	100		100	100

**Table 3:** Percentage distribution of second cognitive debriefing of QOLISSY 0–4 questionnaire (N = 20)

	Strongly disagree	Disagree	Neither agree nor disagree	Agree	Strongly agree
Easy to complete the questionnaire.	-	-	26.7	66.7	6.7
Addressed the everyday challenges of their child related to the diagnosis.	-	-	6.7	80	13.3
Appropriately captured the child’s feelings about living with short stature.	-	-	6.7	66.7	26.7
The questionnaire is complete.	-	-	-	30	70

Additional comments from these cognitive debriefing sessions informed adjustments to the questionnaire, resulting in the inclusion of a new item: “My child is disadvantaged due to the limited accessibility in public spaces.” Other items were reworded as needed for clarity and relevance.

## Discussion

The QoLISSY 0–4 questionnaire was developed to address a critical gap in health-related quality of life (HRQoL) assessment for young children with short stature. Unlike existing tools designed for older children, this PROM captures condition-specific and developmentally relevant experiences from a parental perspective. Its structured domains—spanning physical, social, emotional, and familial dimensions—reflect the everyday realities and challenges faced by children aged 0 to 4 and their caregivers [20].

This age-appropriate tool advances current pediatric HRQoL measurement in two key ways. First, it integrates parent-reported insights on early developmental issues, such as delays in motor milestones and difficulties navigating daycare environments [21, 22]. Second, it provides tailored content to assess how growth-related conditions influence emotional expression, coping, and social inclusion at a formative life stage. In contrast to general instruments like the PedsQL 1–2, QoLISSY 0–4 focuses specifically on the lived experiences associated with short stature, enabling more precise monitoring and intervention planning [23]. By focusing on growth-related physical and psychosocial issues, it offers condition-specific data to inform care decisions for children with short stature, thereby enhancing clinical practice and research by enabling precise monitoring, measuring treatment outcomes, and adapting care plans over time [24].

Building on this framework, cognitive debriefing and pilot testing confirmed that standard HRQoL tools for older children often fail to capture the specific challenges experienced by children aged 0 to 4 with short stature. To address this, the QoLISSY 0–4 questionnaire introduced targeted adaptations across domains such as physical health, social participation, emotional well-being, and coping, guided by detailed parental feedback.

Physical health items were refined to reflect toddler-specific challenges, including fatigue during movement and delayed motor milestones, which restrict opportunities for age-appropriate play and environmental exploration [25, 26]. In the social domain, parents described peer exclusion and daycare-related barriers linked to size, which informed new items on social inclusion and caregiver sensitivity [27, 28].

Emotional responses were also emphasised. Since young children often express distress nonverbally—through tantrums, withdrawal, or stubbornness—the “Emotions and Feelings” domain was revised to include behavioural indicators of anxiety and frustration [29]. Additionally, sleep quality emerged as a significant factor affecting well-being; new items now assess sleep disturbances related to discomfort or mobility limitations [30].

The findings further highlight the dual need to assess both the child’s experiences and the parental caregiving burden. Many parents, especially those new to managing a chronic pediatric condition, reported stress and uncertainty that can indirectly influence the child’s well-being [31]. Capturing these interlinked experiences enables earlier and more comprehensive support.

Clinically, the QoLISSY 0–4 serves as a practical tool for identifying HrQoL concerns during routine care, tailoring interventions, and monitoring developmental progress. It also offers a framework for guiding parent counselling and support, promoting a more responsive and family-centred approach to care in early childhood.

## Limitations

While the QOLISSY 0–4 provides a detailed view into the experiences of young children with short stature, several limitations must be acknowledged. The reliance on parental observations could introduce biases, as parents may interpret or report their child’s behaviours differently [32]. Additionally, our study’s focus on a sample consisting solely of German-speaking parents limits its cultural applicability. Future validation efforts in other languages and cultural settings are essential to enhance the instrument’s generalizability. Cultural differences can significantly influence the perception of HrQoL, and addressing these variations would strengthen the tool’s validity and applicability across diverse populations. Although we employed a rigorous translation process to create an English version, future research should explore the cross-cultural relevance of the instrument, particularly in contexts where short stature may carry different social and medical implications [33].

## Conclusion

The QOLISSY 0–4 questionnaire represents a significant advancement in the measurement of HrQoL for young children with short stature by addressing specific physical, social, and emotional challenges that emerge in early childhood. By focusing on parental perspective and refining items through feedback-driven adjustments, this tool enables a more comprehensive and developmentally appropriate assessment of HrQoL for children aged 0 to 4. This instrument fills a critical gap, supporting early identification of intervention needs and improving the care and support for children and their families during a formative developmental period. Future validation studies across diverse cultural settings will further enhance its robustness and utility, promoting a more tailored approach to well-being assessment in pediatric populations with short stature.

## Data Availability

Data included in this report are available on request.

## Appendix 1

**Table 4:** Cognitive Debriefing for Pilot QOLISSY 0 − 4

**Physische Gesundheit**	
1.1	Mein Kind ist im Allgemeinen gesund.
1.2	Mein Kind leidet unter Schmerzen im Zusammenhang mit seinem Kleinwuchs.
1.3	Mein Kind kann sich altersgemäß bewegen und die Umgebung erkunden.
1.4	Mein Kind ist voller Energie.
1.5	Mein Kind zeigt altersentsprechende feinmotorische Fähigkeiten (z. B. Greifen und Halten von kleinen Gegenständen).
1.6	Mein Kind zeigt altersentsprechende grobmotorische Fähigkeiten (z. B. Krabbeln, Laufen, Springen).
1.7	Mein Kind hat aufgrund seiner Wachstumsprobleme Schwierigkeiten beim Essen.
1.8	Mein Kind hat aufgrund seiner Wachstumsprobleme Schwierigkeiten beim Schlafen.
1.9	Die Wachstumsprobleme meines Kindes beeinträchtigen seine Teilnahme an täglichen Aktivitäten.
**Sozial**	
2.1	Mein Kind nimmt an sozialen Aktivitäten außerhalb der Kindertageseinrichtung teil.
2.2	Mein Kind wurde aufgrund seiner Wachstumsprobleme von anderen Menschen benachteiligt.
2.3	Mein Kind ist in seinen sozialen Aktivitäten eingeschränkt.
2.4	Mein Kind ist in die Familienaktivitäten einbezogen.
2.5	Mein Kind wird von anderen Familienmitgliedern unterstützt.
2.6	Der Wohnraum ist an die körperlichen Fähigkeiten und Bedürfnisse meines Kindes angepasst.
2.7	Mein Kind ist aufgrund der eingeschränkten Barrierefreiheit im öffentlichen Raum benachteiligt.
**Kinderbetreuung**	
3.1	Mein Kind geht gerne in die Kindertageseinrichtung.
3.2	Die Mitarbeitenden der Kindertageseinrichtung kennen sich mit den besonderen Bedürfnissen meines Kindes aus.
3.3	Die Kindertageseinrichtung bietet spezielle Unterstützung für mein Kind mit seinen besonderen Bedürfnissen an.
3.4	Die Ausstattung der Kindertageseinrichtung ist an die Körpergröße meines Kindes angepasst.
3.5	Mein Kind kommt gut mit anderen Kindern in der Kindertageseinrichtung zurecht.
3.6	Andere Kinder in der Kindertageseinrichtung berücksichtigen die besonderen Bedürfnisse meines Kindes.
**Gefühle und Emotionen**	
4.1	Mein Kind ist glücklich und zufrieden.
4.2	Mein Kind ist reizbar.
4.3	Mein Kind ist frustriert.
4.4	Mein Kind zeigt ein altersentsprechendes Verhalten (z. B. Verspieltheit, Neugierde).
4.5	Mein Kind ist gestresst.
4.6	Mein Kind ist ängstlich.
**Zurechtkommen mit Größe**	
5.1	Mein Kind ist frustriert wegen körperlicher Einschränkungen, die mit seinen Wachstumsproblemen zusammenhängen.
5.2	Mein Kind kann die Einschränkungen durch den Kleinwuchs wirksam bewältigen.
5.3	Mein Kind ist selbstbewusst beim Ausprobieren neuer Aktivitäten.
5.4	Mein Kind ist anpassungsfähig und nimmt an den täglichen Aktivitäten teil.
5.5	Mein Kind kann mit Veränderungen positiv umgehen.
**Medizinische Versorgung**	
6.1	Die Häufigkeit der Arztbesuche/Therapeut:innen-Besuche ist anstrengend für mein Kind.
6.2	Mein Kind hat Angst vor Arztbesuchen.
6.3	Mein Kind hat Angst vor den medizinischen Untersuchungen.
6.4	Mein Kind hat Angst vor der Wachstumstherapie.
6.5	Mein Kind hat emotionale Probleme aufgrund der Wachstumstherapie.
6.6	Mein Kind hat körperliche Probleme aufgrund der Wachstumstherapie.
6.7	Medizinische Behandlungen wirken sich negativ auf die Stimmung meines Kindes aus.
6.8	Medizinische Behandlungen wirken sich negativ auf das Verhalten meines Kindes aus.
6.9	Mein Kind hat eine positive Einstellung zur medizinischen Behandlung.
**Zukunft**	
7.1	Ich mache mir Sorgen um die zukünftige Körpergröße meines Kindes.
7.2	Ich mache mir Sorgen um die körperliche Entwicklung meines Kindes.
7.3	Ich mache mir Sorgen über mögliche zukünftige gesundheitliche Komplikationen meines Kindes.
7.4	Ich mache mir Sorgen darüber, ob mein Kind von der Gesellschaft akzeptiert wird, wenn es älter wird.
7.5	Ich mache mir Sorgen darüber, dass mein Kind aufgrund seiner Körpergröße vor schulischen Herausforderungen steht.
7.6	Ich mache mir Sorgen um die Fähigkeit meines Kindes, unabhängig zu werden und sich selbst zu versorgen.
**Einfluss Wachstumsprobleme**	
8.1	Ich fühle mich durch die Wachstumsprobleme meines Kindes emotional überwältigt.
8.2	Ich fühle mich traurig aufgrund der Wachstumsprobleme meines Kindes.
8.3	Ich fühle mich ängstlich aufgrund der Wachstumsprobleme meines Kindes.
8.4	Ich leide unter Müdigkeit und Erschöpfung aufgrund der besonderen Pflegebedürfnisse meines Kindes.
8.5	Die Wachstumsprobleme meines Kindes beeinträchtigen mein Freizeit- und Sozialverhalten.
8.6	Ich fühle mich isoliert aufgrund der Wachstumsprobleme meines Kindes.
8.7	Ich fühle mich unverstanden aufgrund der Wachstumsprobleme meines Kindes.
8.8	Die zusätzliche finanzielle Belastung, die durch die besonderen Bedarfe meines Kindes aufgrund der Wachstumsprobleme bestehen, ist schwer zu bewältigen.
8.9	Ich fühle mich auf zukünftige Herausforderungen aufgrund der Wachstumsprobleme meines Kindes vorbereitet.

## Supplementary quotes from Phase 1 – Cognitive debriefing

Note: The following participant statements are translated and paraphrased excerpts derived from the open-ended cognitive debriefing responses. They are intended to reflect the core sentiments and themes identified during analysis while preserving participant anonymity and readability.

1. Clarity Issues

“I wasn’t entirely sure what ‘participation in physical activities’ was supposed to mean—it could be anything from crawling to climbing at this age.” (Parent #04)

“Some phrases felt like they were written for older children. I had to read them twice to interpret them in the context of my toddler.” (Parent #11)

2. Relevance of Items

“Situations about school or peer pressure don’t apply to our child yet—he’s not even in daycare.” (Parent #08)

“Most of the questions about social settings were hard to answer, because my child mostly interacts with us or close family.” (Parent #16)

3. Importance of Specific Domains

“The emotional side really matters. Our daughter is easily upset when she can’t do what the other kids can.” (Parent #02)

“I appreciated that sleep was included—it’s been a big challenge due to the discomfort he experiences at night.” (Parent #14)

4. Need for Rewording

“Some questions sound too medical. I understood them, but simpler wording would help.” (Parent #17)

“Instead of saying ‘growth-related physical discomfort,’ maybe just say ‘pain because of being small’—that feels more relatable.” (Parent #06)

5. Suggestions for Additional Items

“There should be something about accessibility. Our son can’t use most playground equipment because of his height.” (Parent #10)

“You might include a question about how this affects our daily routines. We always have to plan for extra support or attention when we go out.” (Parent #01)
